# Expanding the evidence base: The impact of Tobacco 21 policies on youth tobacco use

**DOI:** 10.18332/tpc/214471

**Published:** 2026-02-12

**Authors:** Danyi Li, Linyun Fu, Nathan Davies, Steve Sussman, Mary A. Pentz

**Affiliations:** 1 Department of Population and Public Health Sciences, Keck School of Medicine, University of Southern California, Los Angeles, United States; 2 Crown Family School of Social Work, Policy, and Practice, The University of Chicago, Chicago, United States; 3 Nottingham Centre for Public Health and Epidemiology, School of Medicine, Nottingham City Hospital, University of Nottingham, Nottingham, United Kingdom; 4 Department of Psychology, University of Southern California, Los Angeles, United States; 5 Suzanne Dworak-Peck School of Social Work, University of Southern California, Los Angeles, United States

**Keywords:** youth tobacco use, Tobacco 21, meta-analysis, age access law, youth smoking

**Dear Editor,**

The minimum age for tobacco sales was raised to 21 years by the United States federal government in 2019 (known as Tobacco-21 or T21-policy).

Two recent systematic reviews, one with a meta-analysis, have synthesized the effects of T21 and found that T21 may reduce current cigarette-smoking in youth; however, the evidence quality was moderate and did not reach statistical significance^1-3^. The published meta-analysis was on odds ratios, and excluded effects from linear probability models and marginal effects of a unit probability change, both of which are risk differences (RDs)^1,2^. As the evidence on T21 continues to expand, we updated the systematic review and conducted a meta-analysis on RDs to offer a more comprehensive view.

Following the same search strategy and inclusion criteria as a previous meta-analysis^1,2^, we updated the literature search in 14 databases from the last search date, 1 January to 20 October 2025, and identified an additional 405 studies for eligibility screening. We included empirical studies evaluating T21’s effect on current cigarette-smoking in youth (aged 11–20 years) in the US. We constrained the analysis to the effects comparing youth smoking pre- and post-policy in areas with versus without a T21. Four studies, reporting a total of seven effect sizes, were included in a random-effects model^4-7^. Overall, the studies were well-designed and adjusted for key confounders; however, the effects included in the meta-analysis relied on self-reported smoking, which may introduce information and social expectation biases.

As shown in Figure 1, the T21s revealed a one-percentage-point lower risk of current cigarette-smoking (RD= -0.01; 95% CI: -0.03–0.003) compared to the non-T21 areas before policy implementation, although the effect was not statistically significant. The prediction interval (PI) quantifies the range of estimated true effects of future T21 evaluations across different settings^8^. The 95% CI: -0.05–0.02 suggests that for 95% of future studies, the true effects may vary from small increases (2%) to as much as a 5% reduction in current smoking.

**Figure 1:**
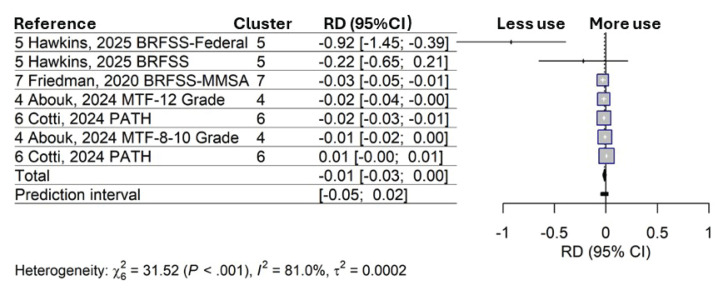
Random-effects model of the effects (risk differences) of Tobacco 21 policies in the United States on past 30-day cigarette smoking in youth aged 11–20 years

Policy adoption does not always lead to reductions in youth smoking; implementation, enforcement, evaluation, and contextual factors must be considered. For example, primacy and recency effects were observed in the Needham 2005 T21 evaluation^9^. The study found a greater decline in youth smoking in Needham than in comparison areas within the first five years following T21 adoption; however, the effect diminished after five years, suggesting a potential floor effect^9^. Policy enforcement strength also plays a role, as shown in one of the recent evaluations on state T21s^10^. Although the study found an overall null policy effect on smoking, further differentiation of policy components revealed that stronger T21s (allowing stricter local policies, requiring retailers to be licensed, and including a violation penalty scheme) were associated with reduced smoking^10^. In addition, T21 may be more effective in areas with higher smoking prevalence and for populations with lower socioeconomic status^3^. Better protection of youth from tobacco use does not solely rely on policy adoption, but also on thoughtful policy design, consistent enforcement, and continuous equity-focused evaluation, as contextual factors and public awareness interact with policies and shape policy effectiveness.

## Data Availability

Data sharing is not applicable to this article as no new data were created.
